# Dopaminergic D1 receptors in the nucleus basalis modulate recovery from propofol anesthesia in rats

**DOI:** 10.22038/IJBMS.2019.37716.8962

**Published:** 2020-03

**Authors:** Ke Li, Yuran Zhou, Bao Fu

**Affiliations:** 1 Department of Anesthesiology, Hospital of Stamatology, Zunyi Medical University, Dalian road, Zunyi 563000, Guizhou, China; 2 Department of Anesthesiology, Affiliated Hospital of Zunyi Medical University, Dalian road 149, Zunyi 563000, Guizhou, China; 3 Department of Critical Care Medicine, Affiliated Hospital of Zunyi Medical University, Dalian road 149, Zunyi 563000, Guizhou, China

**Keywords:** Dopamine D1 receptors, Emergence, Induction, Nucleus basalis, Propofol

## Abstract

**Objective(s):**

Melatonin, an important hormone secreted by the epiphysis, is a powerful anti-oxidant with a high potential to neutralize medical toxins. The goal of this study was to demonstrate the beneficial effect of melatonin on epididymal sperm and reproductive parameters in mice treated with acetylsalicylic acid (ASA).

**Materials and Methods:**

Thirty-nine SD rats were randomly split into D1 receptor agonist (chloro-APB), D1 receptor antagonist (SCH23390), and saline groups after preparing NB microinjection model. We observed the effect of NB microinjection of SCH23390, chloro-APB, or saline on the period of induction and recovery time of propofol anesthesia and recorded the changes of electroencephalogram (EEG) simultaneously.

**Results:**

NB microinjection of chloro-APB accelerated the recovery from propofol anesthesia (*P*<0.05), without affecting the induction of anesthesia (*P*>0.05); NB microinjection of SCH23390 produced the opposite effects. NB microinjection of saline did not influence the induction or recovery of propofol anesthesia (*P*>0.05). NB injection of chloro-APB decreased the ratio of δ power and increasedαand β ratios in prefrontal cortex EEG (*P*<0.05); NB microinjection of SCH23390 increased δ ratio and decreased β ratio (*P*<0.05); NB microinjection of saline had no significant effect on EEG (*P*>0.05)

**Conclusion:**

D1 dopamine receptors in NB are involved in modulating the emergence from propofol anesthesia, but not affecting the induction of propofol anesthesia.

## Introduction

General anesthetics can reversibly cause loss of consciousness (LOC), but the specific mechanism is unclear. In addition to the effects on the cerebral cortex and thalamus (1-4), general anesthetics may induce LOC through the ascending arousal system. Previous research shows that recovery of anesthesia is different from anesthesia induction, and ascending arousal pathways play an important role in regulating the recovery from anesthesia (5). Cholinergic pathway (6), noradrenergic pathway (7), histaminergic pathway (8), and orexinergic (9) pathway have been demonstrated to take part in recovery of anesthesia, while the dopaminergic pathway is still to be explored.

The Nucleus Basalis (NB), which is a part of basal forebrain (BF), is important for activation of the hippocampus and neocortex (10). Damaging of NB cholinergic neurons increases response to general anesthetics (11). Inputs to the BF include noradrenergic fibers from the locus ceruleus (LC), dopaminergic fibers from ventral tegmental area, and the substantia nigra (10). Previous study has demonstrated that norepinephrine injection into NB induces microarousal from desflurane anesthetized rats (12). Whether dopamine receptors in NB are involved in general anesthesia is still unclear. Therefore, this study is to explore the role of dopaminergic D1 receptors in NB in propofol anesthesia. 

## Materials and Methods

All laboratory processes were approved by Zunyi Medical University (Guizhou province, China). Thirty-nine SD male rats (250~300 g, 10~12 weeks old) were purchased from the Army Medical University (Chongqing city, China). Animals were raised and cared for as previously described (13). 

***Rat models of microinjection***

Rats received intraperitoneal injection with sodium pentobarbital (50 mg/kg) and were used to prepare the model of NB microinjection, as previously described (13). A cannula was placed into NB (AP -1.3, ML -2.5, DV 5.0) and an EEG recording electrode was put into PFC (the left prefrontal cortex, AP 3.7, ML 3.2, DV 1.7). The cannula and electrode were immobilized with two small skull screws and dental acrylic. Rats were kept alone to reduce the incidence of catheter displacement. Rats recovered seven days before the next test. 

***Drugs***

D1 agonists (chloro-APB) and D1 antagonists (SCH23390, SigmaAldrich, USA) were soluble in saline (0.9%). The infusion cannula and a tubing were linked to a syringe (10 µl, Hamilton). Drugs were injected using a precision pump (RWD, Shenzhen city, China). Drugs were injected at 0.25 µl/min for 2 min. Animals were divided into four groups: saline, chloro-APB, SCH23390, and chloro-APB+SCH23390 (n=8). 

***Experimental protocol***

In behavioral experiment, the observer did not know what kind of drug is microinjected. Before behavioral experiment, rats were placed in a chamber (RWD Company, Shenzhen, China) for acclimation. We tested the loss and recovery of the righting reflex (LORR and RORR) as previously reported (6, 14, 15).

***Induction of anesthesia***

**Figure 1:**
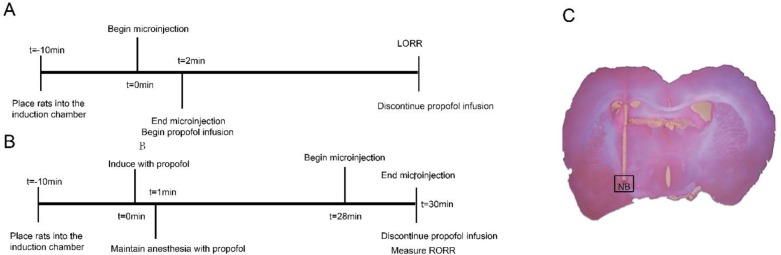
Timelines of microinjection and propofol infusion (A) and schematic of the microinjection site, modified from rat brain atlas (Paxinos& Watson, 2005)

After adapting for 10 min (Figure 1A; t=-10 min), drugs began to infuse to the NB (Figure 1A; t=0 min). After injection, induction of anesthesia started (Figure. 1A; t=2 min) by intravenous infusion of propofol (48 mg/kg/h). The LORR time was calculated as we described previously (13). 

***Recovery from anesthesia***

After acclimation, anesthesia induction started (Figure. 1B; t=0 min) with propofol injection (11 mg/kg) within 30 sec. Anesthetic maintenance started (Figure. 1B; t=1 min) by continuous injection of propofol (48 mg/kg/h). Injection lasted for 30 min (16). Two minutes before stopping propofol injection, rats received an infusion of drugs into the NB (Figure 1B; t=28 min to 30 min). After infusion, rats were placed in supine position. Recovery time was measured as we described previously (13). 

***EEG analyses***

EEG waves were obtained and amplified as previously described (13). Power spectrums were calculated by averaging the signal power (δ: 1- 4 Hz; θ: 4-8 Hz; α: 8-12 Hz; β: 12-25 Hz; and γ: 25-60 Hz).

***Histological localization***

At the end of the test, rats received intraperitoneal injection with sodium pentobarbital (60 mg/kg) and perfused with normal saline and then paraformaldehyde (4%). Brain tissue was soaked in paraformaldehyde (12 hr) and dehydrated in sucrose (30%) for 5 days (4 ^°^C). Blocks (60 µm) including the NB were prepared using a freezing microtome (Leica, Heidelberg, Germany). The sections including NB were placed on a glass slide, then dried and stained as previously described (13).

***Statistical processing***

Statistical processing was performed using GraphPad Prism 5.0 (San Diego, CA, USA). Data were shown as Mean±SD. LORR and RORR time were analyzed by repeated measures one-way ANOVA with *post-hoc* Bonferroni’s correction. *P*<0.05 was considered significant. 

## Results

***Histological localization***

A total of 39 rats were modeled, in which 32 rat cannula sites were located in BF, while 7 rats were not in BF. Each group contained 8 rats. The tissue micrograph of microinjection site was shown in Figure 1C.

***Behavioral results***

**Figure 2:**
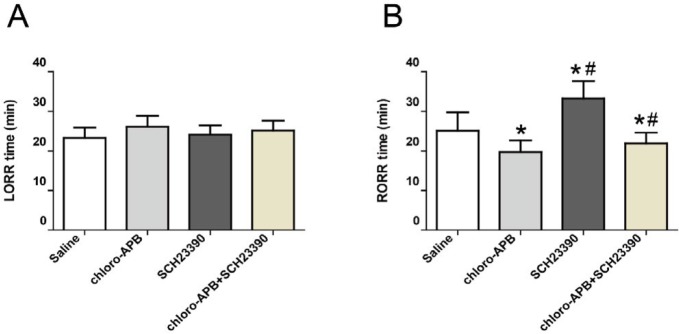
Effects of microinjection of drugs on the induction time (A) and emergence (B) of propofol anesthesia

The LORR time of rats in saline, chloro-APB, SCH23390, and chloro-APB+ SCH23390 groups were 23.31±2.58 min, 26.14±2.76 min, 24.04±2.43 min, and 25.20±2.47 min, respectively, *P*>0.05, n=8, as shown in Figure 2A. 

Compared to the saline group, RORR time of rats in chloro-APB group was significantly shortened (23.16 ±4.66 min *vs* 19.71±2.93 min, *P*<0.05, n=8), while RORR time of rats in the SCH23390 group was markedly prolonged (23.16 ± 4.66 min vs 33.25± 4.32 min, *P*<0.05, n=8). RORR time of rats in the chloro-APB + SCH23390 group was shorter than that in the SCH23390 group, but longer compared with the chloro-APB group (*P*<0.05, n=8, Figure 2B). Our results show that dopamine D1 receptors do not take part in the induction of propofol anesthesia, while they do participate in the recovery of propofol anesthesia.

***EEG changes***

**Figure 3:**
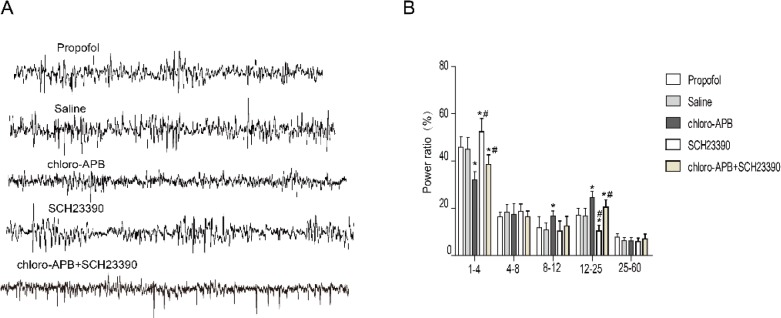
Representative EEG activity traces and corresponding power spectra recorded in the prefrontal cortex (A) and the results of spectral analyses of EEG power (B)

We compared the EEG spectrum of 10 min before and after microinjection. Microinjection of saline had no significant effect on the EEG band (*P>*0.05, n=8). Infusion of chloro-APB decreased the ratio in the δ band (1- 4 Hz, 45.93%±4.31% vs 32.14%±3.36%, *P*<0.05, n=8) in PFC, but increased the power ratio in the α band (8-12 Hz, 11.76%±4.79% *vs* 16.76%±2.14%, *P*<0.05, n=8) and β power (12-25 Hz, 17.18±2.84% *vs* 24.64%±2.54%, *P*<0.05, n=8). However, Infusion of chloro-APB did not affect θ and γ (Figure 3B). 

Comparing with pre-microinjection, infusion of SCH23390 increased the relative power in the δ band (45.93%±4.31% *vs* 52.34%±5.68%, *P*<0.05, n=8) and decreased the β band (17.18±2.84% *vs* 10.46%±2.38%, *P*<0.05, n=8), without changing the relative power in the θ, α, and γ (*P>*0.05, n=8, Figure 3B). 

Compared to pre-microinjection, infusion of chloro-APB +SCH23390 into NB decreased the ratio in the **δ** band and decreased the power ratio in the **β** band (*P*<0.05, n=8), but the degree of change was lower compared with the chloro-APB group (*P*<0.05, n=8). The results demonstrated that infusion of D1 agonist and antagonist not only changed the RORR time of propofol anesthesia, but also induced the EEG changes in PFC. 

## Discussion

General anaesthesia provides analgesia, forgetting, immobility, retardation of autonomic response, and unconsciousness (17, 18), of which loss of consciousness became the research hotspot of anesthesiologists and neuroscientists. Although major progress has been made in the molecular level of general anesthetics, the brain nuclei affected by general anesthetics on which they exert general anesthesia remain unclear. 

Electroencephalogram studies found that propofol induced EEG activity was similar to sleep slow waves and originated from the same cortical area (19). Therefore, sleep-arousal nuclei may take part in the loss of consciousness and emergence induced by anesthetics. The cholinergic arousal system is a vital part of the ascending arousal pathway, which is involved in regulating cortical and behavioral arousal (20). BF, receiving dopaminergic fibers from the ventral tegmental area (VTA) (21), is an important nucleus in the brain that regulates sleep and arousal (22, 23). Therefore, the dopamine receptors in BF may take part in LOC and emergence caused by general anesthetics.

The results showed that injection of D1 receptor agonist into NB did not influence anesthesia induction, while accelerating the emergence from propofol anesthesia. In contrast, D1 receptor antagonists delayed the recovery from propofol anesthesia. The traditional view is that the induction and emergence from general anesthesia are opposite processes, but the results of this study do not support this view. Consistent with our findings, Kelz *et al.* found that the orexin pathway regulates the recovery from isoflurane and sevoflurane anesthesia, while not influencing the induction process (24). Lesion of VTA dopaminergic neurons does not influence induction of propofol and ED50 either, but delays the recovery time (25). Our recent studies demonstrated that injection of norepinephrine into the central thalamus accelerated the recovery of propofol anesthesia in rats, without affecting the induction process (13). These studies suggest that the induction and emergence from general anesthesia may be two independent processes, which mediate by different neural pathways. 

The behavioral arousal is usually associated with the cortical EEG activation. Therefore, we observed the effect of BF microinjection of D1 agonist and antagonist into NB on cortical EEG. We found that microinjection of D1 receptor agonist significantly decreased δ ratio and increased the ratio of α and β waves. Slow-δ and α oscillations are markers of propofol-induced unconsciousness (26). Similar to our results, microinjection of norepinephrine into the central medial thalamus significantly reduced the δ band and increased α ratio in the anterior cingulate cortex (13). Infusion of norepinephrine into BF caused microarousal in rats under desflurane anesthesia, and the ratio of δ in prefrontal cortex decreased significantly (12). In this study, microinjection of D1 agonist into NB not only accelerated the emergence from propofol anesthesia, but also triggered EEG activation in the prefrontal cortex. In addition, we also assessed the effect of co-administration of chloro-APB and SCH23390. We found that co-administration of chloro-APB and SCH23390 also have the effect of arousal, but the effect is lower than that of chloro-APB. We speculate that microinjection of D1 receptor agonist may activate D1 receptors on cholinergic neurons and glutamatergic neurons in NB, inducing the increase of acetylcholine and glutamate release and causing cortical arousal.

A previous research demonstrated that methylphenidate causes emergence from isoflurane anesthesia (27) and droperidol suppresses methylphenidate-induced arouse. However, since droperidol suppresses both adrenergic and dopaminergic receptors, the specific mechanisms responsible for methylphenidate are still unclear. Our study shows that activating D1 receptors is enough to cause recovery from propofol anesthesia. A previous study reported that chloro-APB is a respiratory stimulant (28), so it may be that chloro-APB induced a decrease in duration to recovery of isoflurane anesthesia by a combination of increased arousal and minute ventilation. The propofol used in this study is an intravenous anesthetic and is not affected by minute ventilation. Therefore, the results of the present study indicate that activating D1 dopamine receptors can accelerate recovery from propofol anesthesia and cause cortical arousal.

The following are the limitations of this study. Firstly, this study only observed the effect of dopamine D1 receptors on the induction and emergence from propofol anesthesia, while effects of D2 receptors were not observed. Secondly, although we observed that microinjection of D1 receptor agonists accelerated the emergence from propofol anesthesia, we did not explore the intracellular signaling mechanisms triggered by D1 receptors. 

## Conclusion

D1 dopamine receptors in NB are involved in modulating the emergence from propofol anesthesia, but do not affect the induction of propofol anesthesia.

## References

[1] Vahle-Hinz C, Detsch O, Siemers M, Kochs E (2007). Contributions of GABAergic and glutamatergic mechanisms to isoflurane-induced suppression of thalamic somatosensory information transfer. Exp Brain Res.

[2] Velly LJ, Rey MF, Bruder NJ, Gouvitsos FA, Witjas T, Regis JM (2007). Differential dynamic of action on cortical and subcortical structures of anesthetic agents during induction of anesthesia. Anesthesiology.

[3] Fu B, Wang Y, Yang H, Yu T (2016). Effects of etomidate on GABAergic and glutamatergic transmission in rat thalamocortical slices. Neurochem Res.

[4] Fu B, Liu C, Zhang Y, Fu X, Zhang L, Yu T (2017). Ketamine attenuates the glutamatergic neurotransmission in the ventral posteromedial nucleus slices of rats. BMC Anesthesiol.

[5] Taylor NE, Chemali JJ, Brown EN, Solt K (2013). Activation of D1 dopamine receptors induces emergence from isoflurane general anesthesia. Anesthesiology.

[6] Alkire MT, McReynolds JR, Hahn EL, Trivedi AN (2007). Thalamic microinjection of nicotine reverses sevoflurane-induced loss of righting reflex in the rat. Anesthesiology.

[7] Zhang Y, Fu B, Liu C, Yu S, Luo T, Zhang L (2019). Activation of noradrenergic terminals in the reticular thalamus delays arousal from propofol anesthesia in mice. FASEB J.

[8] Luo T, Leung LS (2011). Involvement of tuberomamillary histaminergic neurons in isoflurane anesthesia. Anesthesiology.

[9] Zecharia AY, Nelson LE, Gent TC, Schumacher M, Jurd R, Rudolph U (2009). The involvement of hypothalamic sleep pathways in general anesthesia: testing the hypothesis using the GABAA receptor beta3N265M knock-in mouse. J Neurosci.

[10] Leung LS, Luo T, Ma J, Herrick I (2014). Brain areas that influence general anesthesia. Prog Neurobiol.

[11] Leung LS, Petropoulos S, Shen B, Luo T, Herrick I, Rajakumar N (2011). Lesion of cholinergic neurons in nucleus basalis enhances response to general anesthetics. Exp Neurol.

[12] Pillay S, Vizuete JA, McCallum JB, Hudetz AG (2011). Norepinephrine infusion into nucleus basalis elicits microarousal in desflurane-anesthetized rats. Anesthesiology.

[13] Fu B, Yu T, Yuan J, Gong X, Zhang M (2017). Noradrenergic transmission in the central medial thalamic nucleus modulates the electroencephalographic activity and emergence from propofol anesthesia in rats. J Neurochem.

[14] Franks NP (2008). General anaesthesia: from molecular targets to neuronal pathways of sleep and arousal. Nat Rev Neurosci.

[15] Hudetz AG, Vizuete JA, Pillay S (2011). Differential effects of isoflurane on high-frequency and low-frequency gamma oscillations in the cerebral cortex and hippocampus in freely moving rats. Anesthesiology.

[16] Dutta S, Matsumoto Y, Gothgen NU, Ebling WF (1997). Concentration-EEG effect relationship of propofol in rats. J Pharm Sci.

[17] Rudolph U, Antkowiak B (2004). Molecular and neuronal substrates for general anaesthetics. Nat Rev Neurosci.

[18] Campagna JA, Miller KW, Forman SA (2003). Mechanisms of actions of inhaled anesthetics. N Engl J Med.

[19] Murphy M, Bruno MA, Riedner BA, Boveroux P, Noirhomme Q, Landsness EC (2011). Propofol anesthesia and sleep: a high-density EEG study. Sleep.

[20] Alkire MT, Hudetz AG, Tononi G (2008). Consciousness and anesthesia. Science.

[21] Gaykema RP, Zaborszky L (1996). Direct catecholaminergic-cholinergic interactions in the basal forebrain Substantia nigra-ventral tegmental area projections to cholinergic neurons. J Comp Neurol.

[22] Fuller PM, Sherman D, Pedersen NP, Saper CB, Lu J (2011). Reassessment of the structural basis of the ascending arousal system. J Comp Neurol.

[23] Xu M, Chung S, Zhang S, Zhong P, Ma C, Chang WC (2015). Basal forebrain circuit for sleep-wake control. Nat Neurosci.

[24] Kelz MB, Sun Y, Chen J, Cheng Meng Q, Moore JT, Veasey SC (2008). An essential role for orexins in emergence from general anesthesia. Proc Natl Acad Sci U S A.

[25] Zhou X, Wang Y, Zhang C, Wang M, Zhang M, Yu L (2015). The role of dopaminergic VTA neurons in general anesthesia. PLoS One.

[26] Purdon PL, Sampson A, Pavone KJ, Brown EN (2015). Clinical electroencephalography for anesthesiologists: Part I: background and basic signatures. Anesthesiology.

[27] Solt K, Cotten JF, Cimenser A, Wong KF, Chemali JJ, Brown EN (2011). Methylphenidate actively induces emergence from general anesthesia. Anesthesiology.

[28] Lalley PM (2004). Dopamine1 receptor agonists reverse opioid respiratory network depression, increase CO2 reactivity. Respir Physiol Neurobiol.

